# Chitosan Surface-Modified PLGA Nanoparticles Loaded with Cranberry Powder Extract as a Potential Oral Delivery Platform for Targeting Colon Cancer Cells

**DOI:** 10.3390/pharmaceutics15020606

**Published:** 2023-02-10

**Authors:** Mona M. Mostafa, Maha M. Amin, Mohamed Y. Zakaria, Mohammed Abdalla Hussein, Marium M. Shamaa, Shady M. Abd El-Halim

**Affiliations:** 1 Department of Pharmaceutics and Industrial Pharmacy, Faculty of Pharmacy, October 6 University, 6th of October City, Giza 12585, Egypt; 2 Department of Pharmaceutics and Industrial Pharmacy, Faculty of Pharmacy, Cairo University, Cairo 11562, Egypt; 3 Department of Pharmaceutics and Industrial Pharmacy, Faculty of Pharmacy, Port Said University, Port Said 42526, Egypt; 4 Department of Pharmaceutics and Industrial Pharmacy, Faculty of Pharmacy, King Salman International University, Ras Sudr 46612, South Sinai, Egypt; 5 Faculty of Applied Health Sciences Technology, October 6 University, 6th of October City, Giza 12585, Egypt; 6 Biochemistry Department, Clinical and Biological Sciences Division, College of Pharmacy, Arab Academy for Science, Technology and Maritime Transport, Alexandria 1029, Egypt

**Keywords:** cranberry powder extract, colon cancer, chitosan surface-modified PLGA nanoparticles, HT-29 cell line, cell targeting

## Abstract

Nutraceutical cranberry powder extract (CBPE) has distinct polyphenols inhibiting colon cancer growth and proliferation. However, its oral therapeutic efficacy is hindered because of its low permeability. This study aims to formulate chitosan surface-modified PLGA nanoparticles (CS-PLGA NPs) for encapsulating CBPE and modulating its release rate, permeation, cell targeting, and, therefore, its cytotoxicity. A full 2^3^ factorial design is employed to scrutinize the effect of lactide/glycolide ratio, PLGA weight, and stabilizer concentrations on entrapment efficiency percentage (EE%), particle size (PS), polydispersity index (PDI), and zeta potential (ZP). The optimum formula (F4) shows spherical particles with a relatively high EE% (72.30 ± 2.86%), an appropriate size of 370.10 ± 10.31 nm, PDI; 0.398 ± 0.001, and ZP; −5.40 ± 0.21 mV. Alongside the ATR-FTIR outcomes, the chitosan surface-modified formula (CS-F4) demonstrates a significant increase in particle size (417.67 ± 6.77 nm) and a shift from negative to positive zeta potential (+21.63 ± 2.46 mV), confirming the efficiency of surface modification with chitosan. The intestinal permeability of F4 and CS-F4 is significantly increased by 2.19- and 3.10-fold, respectively, compared to the CBPE solution, with the permeability coefficient (*P*_app_) being 2.05 × 10^−4^ cm/min and 2.91 × 10^−4^ cm/min, for F4 and CS-F4, respectively, compared to the CBPE solution, 9.36 × 10^−5^ cm/min. Moreover, CS-F4 evidences significant caspase-3 protein level expression stimulation and significant inhibition of vascular endothelial growth factor (VEGF) and signal transducer and activator of transcription-3 (STAT-3) protein expression levels, confirming the superiority of CS-F4 for targeting HT-29 cells. Briefly, CS-PLGA NPs could be regarded as a prosperous delivery system of CBPE with enhanced permeation, cell targeting, and antitumor efficacy.

## 1. Introduction

Cancer is considered one of the most violent and disastrous diseases, threatening millions of individuals and increasing their mortality rate. The term “cancer” refers to the uncontrolled growth and multiplication of cells, characterized by the insensitivity to anti-growth signals. These cells have eluded apoptosis with a limitless replication capacity, produce angiogenesis, and, finally, metastasis [1].

Colorectal cancer (CRC) is ranked as the third most prevalent disease globally and one of the most destructive forms of cancer [2]. Several factors, including genetics, lifestyle, diet, and the immune system, are related to colon cancer etiology. Genetic, pharmacological, and epidemiological studies demonstrate the correlation between inflammation and colon cancer [3]. CRC affects the lining of epithelial cells in the colon’s lumen, and the degree of invasion, lymph node metastasis, and distant metastasis characterize its progression. Consequently, CRC management is greatly influenced by the tumor’s characteristics and the patient’s response [2].

Multiple CRC therapy techniques are now in use; however, no strategies exist to suppress this disease entirely because cancer treatment is generally restricted to surgery, radiation, and chemotherapy, all of which have limitations and do not guarantee adequate outcomes. The drugs’ poor solubility in aqueous media, non-specific targeting, drug resistance, low retention effectiveness, and adverse effects on normal cells are among the restrictions for CRC management. As a result, novel therapeutic strategies are always necessary to enhance the effectiveness and safety of cancer treatment [4].

In cell line and animal studies, bioactive components from plant-based foods, particularly vegetables, fruits, and whole grains, have effectively protected against colon cancer [3]. Cranberries (*Vaccinium macrocarpon* Aiton) are rich in polyphenols, which include three classes of flavonoids (flavonols, anthocyanins, and proanthocyanidins), hydroxycinnamic, catechins, triterpenoids, and a variety of phenolic acids that provide antioxidant, enzyme activity alteration, and gene-expression regulating effects, as well as antiviral, antibacterial, anticarcinogenic, antimutagenic, antitumorigenic, antiangiogenic, and anti-inflammatory activities [5,6].

Cranberry powder extract (CBPE) is rich in polyphenols, which are powerful antioxidants that can scavenge free radicals and minimize cellular damage. Polyphenols’ antioxidant mechanism comes from their direct action, neutralizing the reactive oxygen species and eradicating the superoxide free radicals [6,7]. In addition, they interfere indirectly with cell signaling, as polyphenols can trigger the transcription factor NF-E2-related factor 2 (Nrf2), which is essential for the modulation of antioxidant enzymes’ expressions such as glutathione and catalase.

Furthermore, in colon carcinogenesis, pro-inflammatory cytokines are significantly reduced by CBPE treatment. It achieves this by modulating the signaling pathways and proteins involved in inflammation, cell proliferation, and apoptosis in colon cancer. As a result, CBPE could amend the expression of stress-responsive genes, causing an increase in the endogenous antioxidant response [6,8,9]. Despite its therapeutic efficacy, CBPE is a water-soluble extract known to be quickly removed from plasma because of how rapidly it is metabolized and cleared by the kidneys. Its limited intestinal permeability also contributes to its low therapeutic efficacy [10].

The fabrication of medicated nanoparticles has emerged as a viable multifaceted strategy with significant anticancer management activity. The development of tumor-targeting nanoparticles that may release the encapsulated bioactive compounds following the biological parameters of the tumor microenvironment has attracted researchers’ interest. These nanocarriers can enhance the drugs’ bioavailability, their accumulation at the tumor site, and tumor cellular uptake, thereby enhancing their antitumor effects [11].

The use of biodegradable polymers in polymeric nanoparticles (PNPs) fabrication has shown considerable promise to increase the chemotherapeutic efficacy of anticancer drugs by enhancing their delivery. They offered advantages over nonbiodegradable polymers in terms of safety, eliminating the need to be removed from the body, and in being nontoxic and non-immunogenic. In addition, PNPs, as a drug delivery system, offer numerous advantages, including the ability to modify drug release profiles, protect pharmaceuticals from chemical and enzymatic deterioration, and also provide intracellular drug transportation via passive and active targeted properties to specific organs such as the colon via the surface modification approach [12,13,14,15]. Compared to other colloidal systems, like liposomes and polymeric micelles, they exhibit excellent stability, better controllable physicochemical features, more homogeneous size distribution, higher drug payload, and optimum controlled drug release behavior via diffusion through the polymeric matrix or by erosion and degradation of the particles [16,17].

Poly (d,l-lactide-co-glycolide) (PLGA) is among the most widely exploited biocompatible and biodegradable polymers the FDA has approved for both hydrophilic and hydrophobic drug delivery [18,19] since its hydrolysis yields a nontoxic oligomer and monomer of lactic and glycolic acids and is eventually removed as water and carbon dioxide [20].

Variations in the lactic acid and glycolic acid ratios during PLGA polymerization result in its formulation with a wide range of molecular weights, viscosities, and physicochemical characteristics, all of which have a direct impact on PNPs properties like particle size, encapsulation efficiency, drug release strengths, and bioavailability [21,22].

Meanwhile, nanoparticle surface charges also profoundly impact how they interact with cells and their uptake [20]. PLGA nanoparticles (PLGA NPs) have a slightly negative surface charge, which limits their interaction with the negatively charged cell surfaces, resulting in reduced intracellular uptake [19,23]. In an attempt to solve this problem, the surface charge of the prepared PLGA NPs was altered by coating them with chitosan (CS), a naturally occurring cationic polysaccharide that is nontoxic, biodegradable, and biocompatible [23].

The positively charged nanoparticles permit a significant degree of internalization because of the ionic interactions among positively charged nanoparticles and negatively charged cell membranes. Furthermore, after internalization, positively charged nanoparticles appear to have the ability to escape from lysosomes and possess perinuclear localization, whereas neutrally and negatively charged nanoparticles favor colocalizing with lysosomes [20].

To the best of our knowledge, the encapsulation of CBPE into polymeric nanoparticles for the targeting of colon cancer has not been investigated yet. Based on the preceding, the current research strives to boost the intestinal permeation, cellular uptake, targeting, and cytotoxicity of CBPE against colon cancer cells by developing and optimizing promising CBPE-loaded PLGA NPs (CBPE-PLGA NPs) as a potential delivery platform using the double emulsion solvent evaporation technique and chitosan as a surface modifier. To accomplish our goal, the prepared PLGA NPs were physically characterized based on their average particle size, entrapment efficiency, zeta potential, polydispersity index, and morphology. In addition, the optimized CBPE-PLGA NPs were further assessed using attenuated total reflection–Fourier transform infrared (ATR-FTIR) spectroscopy and transmission electron microscopy (TEM) to investigate the structural characteristics of the synthesized nanoparticles and assess the efficiency of the surface modification. Furthermore, the enhancement of the intestinal permeability derived by the optimized formulations was assessed using an ex vivo non-everted rat intestinal sac model. The cytotoxicity of the optimized PLGA NPs was assessed against human colon cancer cell lines, HT-29, through caspase-3, vascular endothelial growth factor (VEGF), and signal transducer and activator of transcription-3 (STAT-3) proteins levels assay. The cytotoxic activity was assessed compared to the CBPE solution.

## 2. Materials and Methods

All details concerning materials, kits, cell line, and instruments used throughout the work are cited in the Supplementary Materials.

### 2.1. Statistical Design and Optimization of CBPE-PLGA NPs

A 2^3^ full factorial design was applied to study the effects of lactide/glycolide (L/G ratio; X1), PLGA weight (PLGA wt; X2), and PVA concentration in the internal aqueous phase (IAP; X3) of the fabricated CBPE-PLGA NPs on the dependent variables: entrapment efficiency percentage (EE%; Y1), mean particle size (PS; Y2), polydispersity index (PDI; Y3), and zeta potential (ZP; Y4).

Each factor was assigned at two levels, as shown in Table 1. All data were evaluated using ANOVA, with a significance value set at (*p* < 0.05). Following statistical analysis, the desirability value was estimated using Design Expert^®^ software (version 7; Stat-Ease, Inc., Minneapolis, MN, USA) to select the optimum formula.

### 2.2. Preparation of CBPE-PLGA NPs

The modified double emulsion (W/O/W) solvent evaporation method was used to fabricate the different CBPE-PLGA NPs [18]. The composition of the different prepared CBPE-PLGA NPs is given in Table 2. Briefly, the specified amount of PLGA polymer was dissolved in 3 mL of dichloromethane (DCM) to prepare the organic phase (OP). To form the internal aqueous phase (IAP), the water-soluble CBPE was dissolved in 10 mL of (deionized water or 1% *w*/*v* aqueous PVA solution). After that, the IAP was added dropwise to the OP via a syringe needle of 22 G, followed by probe sonication for 5 min (5 s on, 2 s off, and the amplitude was set at 50%) in an ice bath to produce the primary emulsion (W/O). The prepared primary emulsion was additionally emulsified by adding it portion-wise to 30 mL of 1% *w*/*v* aqueous PVA solution as an external aqueous phase (EAP) using probe sonication as previously mentioned. The formed double emulsion (W/O/W) was left at 25 °C on a magnetic stirrer (600 rpm) for 3 h to evaporate the organic solvent. Any remaining DCM was then eliminated under reduced pressure using a rotary evaporator.

### 2.3. In Vitro Characterization of the Prepared CBPE-PLGA NPs

#### 2.3.1. Calculation of EE%

The CBPE EE% in the variously prepared CBPE-PLGA NPs was calculated by determination of the unentrapped CBPE indirectly using the ultrafiltration-centrifugation technique described by Soliman et al. [10]. The unentrapped CBPE was measured with a UV/VIS spectrophotometer at λ_max_ 280 nm. The EE percentage of CBPE was determined using the following equation:

EE (%) = (Initial CBPE amount − Free CBPE) / (Initial CBPE amount) × 100(1)

#### 2.3.2. Estimation of PS, PDI, and ZP

The PS, PDI, and ZP were evaluated using a Malvern Zetasizer. Before measurement, the developed CBPE-PLGA-NPs were 100-fold diluted using deionized water and then slightly shaken [24].

### 2.4. Optimization of the Prepared CBPE-PLGA NPs

The desirability was adjusted to achieve the optimum formula by assuming the highest EE% and smallest PS giving the highest priority to EE% while keeping the ZP and PDI values within their ranges. The formula owing the greatest desirability value was selected as the optimum formula. To validate the efficacy of this design, the agreement between the predicted and adjusted outcomes was assessed.

### 2.5. Surface Modification of the Optimum CBPE-PLGA NPs Formula Using Chitosan

The preparation procedure, as mentioned earlier, were applied with minor modifications to prepare chitosan surface-modified PLGA NPs (CS-CBPE-PLGA NPs) from the optimum formula. Chitosan (CS) was dissolved in 1% *w*/*v* acetic acid to prepare a chitosan solution. After that, to prepare both IAP and EAP, the chitosan solution was mixed with PVA solution to make a solution containing 1% chitosan and 1% PVA. The pH of the different aqueous phases was adjusted to 4.5 using 1 M sodium hydroxide [25,26]. CS-CBPE-PLGA NPs were characterized in terms of EE%, PS, PDI, and ZP, as previously mentioned.

### 2.6. In Vitro Characterization of the Optimized CBPE-PLGA and CS-CBPE-PLGA NPs

#### 2.6.1. Transmission Electron Microscopy (TEM)

TEM was applied to explore the morphology of the optimized CBPE-PLGA NPs and CS-CBPE-PLGA NPs using the method described by Zakaria et al. [27].

#### 2.6.2. Attenuated Total Reflection–Fourier Transform Infrared (ATR-FTIR)

The structural modifications of pure CBPE, CBPE-PLGA NPs, and plain PLGA NPs of the optimum formula, together with pure CS and CS-CBPE-PLGA NPs, were investigated using ATR-FTIR spectroscopy, according to the method outlined by Soliman et al. [28].

#### 2.6.3. In Vitro Release Study

The method described by Weng et al. [29] was used to study the release profile of CBPE from the optimized CBPE-PLGA NPs and CS-CBPE-PLGA NPs; 1 mL from each formulation (equivalent to 30 mg of CBPE) was deposited in a beaker that contained 19 mL of phosphate buffer (0.1 M, pH 6.8). The temperature was held at 37 ± 0.5 °C, whereas the shaking water bath was constantly shaken at 100 rpm. At certain time intervals up to 24 h, 1 mL aliquots were withdrawn, the suspensions were centrifuged at (20,000 rpm, 5 min), and the clear supernatant was collected for CBPE analysis using a UV/VIS spectrophotometer at λ_max_ 280 nm. The residue containing NPs was reintroduced to the dissolution media after resuspension in 1 mL fresh medium of phosphate buffer (0.1 M, pH 6.8).

A model-independent parameter, the similarity factor “*f*_2_”, is proposed to determine whether the difference between the release profiles is significant using the equation stated by Amin et al. [30]. If the “*f*_2_” value is between 50 and 100, the data of two release profiles are likely to be identical. Mathematical studies based on zero order, first order, second order, Higuchi, and Korsmeyer–Peppas models were utilized to examine the release profile data [31].

#### 2.6.4. Ex Vivo Permeation Study

The effectiveness of CBPE permeating via the intestinal mucosa from the optimized CBPE-PLGA NPs and CS-CBPE-PLGA NPs compared to the CBPE solution was assessed using the non-everted rat intestinal sac model. This model is considered as a valuable method for investigating the ex vivo drug absorption mechanisms because of the involvement of transporters and intestinal enzymes in drug absorption and transport through the gut, which is not the case upon performing in vitro drug release studies [32].

The intestinal mucosal permeation test was permitted via the Research Ethics Committee (REC), Faculty of Pharmacy, Cairo University (# PI 2840). Male Wistar rats (200–250 g) were housed in a clean cage with adequate food and water supply. Rats were starved overnight and given access to water. They were then sacrificed via cervical dislocation while anesthetized with thiopental, and the small intestines were dissected. The small intestines were adequately cleansed with phosphate buffer saline (PBS) (0.1 M, pH 6.8) and cut into 4 cm long segments. The segments were loaded separately with 1 mL of the prepared PLGA NPs equivalent to 30 mg of CBPE. Before placing the intestine segments in beakers containing 19 mL of PBS (0.1 M, pH 6.8), both ends were carefully tied to avoid seepage. The receptor phase was kept at 37 ± 0.5 °C while being constantly shaken at 100 rpm with the aid of a shaking water bath [33]. A CBPE solution was used to compare the data.

Samples were taken at different intervals over 24 h, diffused through a syringe Millipore filter (0.4 µm), and evaluated using HPLC at a λ_max_ of 280 nm, based on Casanella et al.’s method [34]. The amount of CBPE permeated was calculated by measuring the surface area of the intestinal sac, which was modeled as a cylinder [35]. The apparent permeability coefficient (Papp) was computed using the equation declared by Feng et al. [36].

#### 2.6.5. Stability Study

The optimum CBPE-PLGA NPs and CS-CBPE-PLGA NPs dispersions were kept at 4 °C for 3 months in tightly closed vials. Samples were collected at the beginning and end of the storage period. The influence of storage on EE%, PS, ZP, PDI, and the overall appearance of the stored PLGA NPs dispersions was examined and assessed statistically utilizing GraphPad InStat^®^ software (version 3; GraphPad Software, Inc., San Diego, CA, USA) using Student’s *t*-test. The significance of the results was compared at level (*p* < 0.05) [37].

### 2.7. Cell-Based Antitumor Activity

#### 2.7.1. MTT Cytotoxicity Assay

Cranberry powder extract solution, the optimum CBPE-PLGA NPs, and CS-CBPE-PLGA NPs were screened against colon cancer cell lines, named HT-29, using Sigma in vitro assay kit, MTT based (Sigma-Aldrich Chemical Co., St Louis, MO, USA) [38]. HT-29 cells were plated in a complete growth medium (100 µL) + each compound (100 µL) per well in a 96-well plate for 72 h. Cytotoxic activity was measured using MTT assay using the manufacturer’s protocol. Absorbance was measured spectrophotometrically at λ_max_ = 570 nm, and IC_50_ (inhibition concentration) values were calculated.

#### 2.7.2. Caspase 3 Protein Level Assay

Induction of apoptosis was measured using a caspase-3 activation assay kit (Abcam, Cambridge, UK, Catalog # ab281235) to quantify the level of active caspase-3 proteins according to the manufacturer’s protocol [39,40]. Kit reagents were added following the protocol, and the absorbance was measured at 405 nm using an ELISA reader (Belgium, Europe).

#### 2.7.3. Vascular Endothelial Growth Factor (VEGF) Protein Level Assay

Growth inhibition activity was measured using a VEGF assay kit (Abcam, Cambridge, UK, Catalog # ab209882) to quantify the level of VEGF proteins according to the manufacturer’s protocol [41]. Kit reagents were added following the protocol, and the absorbance was measured at 405 nm using an ELISA reader.

#### 2.7.4. Signal Transducer and Activator of Transcription-3 (STAT-3) Assay

Growth inhibition activity was measured using a STAT-3 assay kit (Abcam, Cambridge, UK, Catalog # ab126459) to quantify the level of STAT-3 proteins according to the manufacturer’s protocol [42]. Kit reagents were added following the protocol, and the absorbance was measured at 405 nm using an ELISA reader.

## 3. Results and Discussion

### 3.1. Factorial Design Outcomes

**Figure 1:**
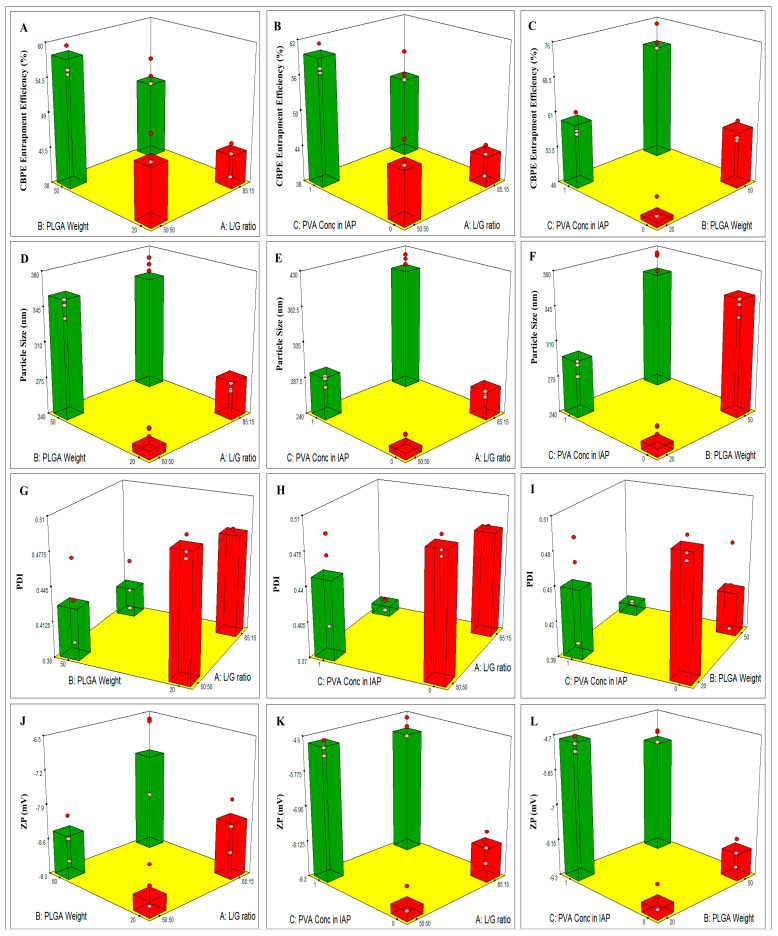
3D plots illustrating the effect of L/G ratio, PLGA weight, and PVA concentration in IAP on (**A**–**C**) CBPE EE%, (**D**–**F**) PS, (**G**–**I**) PDI, and (**J**–**L**) ZP.

Table 3 depicts the design analysis outcomes of the effect of each of the three different factors individually. The predicted and adjusted (R^2^) outcomes agreed with each other. It showed that a preferable ratio of adequate precision (>4) was observed in all the investigated responses. Figure 1 clarifies the influence of the various independent variables, including L/G ratio (X_1_), PLGA wt (X2), and PVA concentration in IAP (X3) on the EE% (Y1), PS (Y2), PDI (Y3), and ZP (Y4) of CBPE-PLGA NPs, as discussed below.

#### 3.1.1. Influence of Formulation Variables on EE% (Y1)

In PLGA nanoparticles, EE% values are correlated to the solubility of the entrapped substance in water. PLGA exhibits a propensity for encapsulating hydrophobic pharmaceuticals more efficiently. Hence, it is difficult to encapsulate a hydrophilic drug as it tends to rapidly penetrate from the inner aqueous phase to the external aqueous phase during the formation of the PLGA NPs via the double emulsion solvent evaporation method, resulting in a lower EE% [22,43]. The ability of the prepared PLGA NPs to encapsulate a large quantity of water-soluble CBPE posed a significant obstacle. The EE% values of the prepared CBPE-PLGA NPs ranged from 41.95 ± 2.84% to 72.30 ± 2.86%, as shown in Table 2.

The EE% was significantly influenced by L/G ratio (X1, *p* < 0.0001). CBPE’s entrapment efficiency values increased as the L/G ratio decreased. The %EE could be attributed to the glass transition temperature (*T_g_*) of the PLGA polymer, which is the temperature at which an amorphous polymer acquires the glassy-state characteristics of stiffness, brittleness, and rigidity upon cooling [44]. The reported *T_g_* for PLGA 50:50 and PLGA 85:15 are 45–50 °C and 50–55 °C, respectively, which indicates that as the L/G ratio grows, *T_g_* increases [45]. *T_g_* and polymer molecular weight are positively correlated. As polymer chains lengthen, the chain end concentration per unit volume decreases, resulting in less free volume between chain ends and a consequent increase in the *T_g_* [46].

The development of glassy PLGA via solvent extraction (removal) is equivalent to a temperature reduction (cooling) below *T_g_*, where the polymer starts to precipitate [47]. In their glassy state, PLGA polymer chains experience slow relaxation to attain a thermodynamic equilibrium glassy state. This gradual change beneath *T_g_* to equilibrium is referred to as physical aging, where the polymer becomes more energetically stable. It means that the polymer with the lowest *T_g_* value (PLGA 50:50) will reach this state in a short time and implies rapid precipitation with the complete formation of an overall stable chain network structure that entraps a large amount of the dissolved drug compared to the higher *T_g_* polymer (PLGA 85:15), which requires more time to reach the state of equilibrium [48].

The EE% increased significantly when the PLGA wt (X2) was increased (*p* < 0.0001). The existence of a higher polymer wt promotes the drug–polymer interaction, which promotes more extract encapsulation [49]. Furthermore, increasing the particle size with the increase in PLGA wt expands the length of the diffusional pathways of drugs to the external aqueous phase, decreases the drug loss through diffusion, and thereby enhances its entrapment [50]. The increase in PLGA wt increases the organic phase viscosity, the barrier between the two aqueous phases [51], which increases drug molecules’ diffusional resistance to the outer aqueous phase. As a result, the drug leakage was minimized, boosting the drug entrapment within the polymeric structure [52].

The abovementioned result was also achieved by incorporating 1% *w*/*v* PVA in the internal aqueous phase (X3), where the EE% was significantly increased (*p* < 0.0001). The incorporation of PVA increases the IAP viscosity, enhances CBPE retention, and prevents leaching from the prepared PLGA NPs [43,53].

#### 3.1.2. Influence of Formulation Variables on PS (Y2)

The size of the PLGA NPs influences their interaction with cell membranes and their cellular uptake. PLGA NPs lie in the range from 50 to 500 nm, generally suitable for cellular uptake via endocytosis [43]. In this study, CBPE-PLGA NPs’ average PS varied between 259.87 ± 4.68 nm and 423.10 ± 8.36 nm (Table 2), and accordingly, they could be endocytosed.

The first point that stands out is that PS increased significantly (*p* < 0.0001) in the PLGA-85:15-based formulae as the monomer L/G ratio increased. As previously mentioned, by increasing the L/G ratio, *T_g_* increases [45]. Polymer molecular weight (Mwt) and *T_g_* positively correlate [46]_._ Therefore, the PLGA polymer with a higher L/G ratio has a higher Mwt and, thus, a higher inherent viscosity. The Mwt of PLGA 85:15 is reported to be 50.000–75.000 with an inherent viscosity of 0.55–0.75 dL/g, and the Mwt of PLGA 50:50 is 24.000–38.000 with an inherent viscosity of 0.32–0.44 dL/g. Therefore, PLGA 85:15 produces an organic phase with a higher viscosity, lowering the net shear stress for droplet breakdown. As a result, it produces PNPs larger in size compared to PLGA 50:50, which produces organic phase droplets with low viscosity that break more easily during sonication. Accordingly, PLGA 50:50 produces smaller, average-sized nanoparticles [54,55].

In addition, due to the decreased hydrophobic integrity of PLGA 50:50, the organic solvent nano-droplets containing PLGA 50:50 escape faster, resulting in a quicker solidification/precipitation of the formulated PLGA NPs, which reduces the possibility of droplet adhesion and fusion during solvent evaporation and contributes to lower particle sizes [55].

Another noteworthy point is that PLGA wt (X2) had a significant influence on PS results (*p* < 0.0001). According to the results, the particle size increased with increasing the polymer wt, which might probably be due to the high PLGA content within the PLGA NPs shell [56]. It could also be clarified by considering the number of polymer chains per unit volume of organic solvent and its viscosity. So, by increasing PLGA wt, the organic phase’s viscosity was increased. Consequently, a lower net shear stress is implied, as discussed previously, resulting in resisting droplet breakdown, thereby forming larger droplets [52,57].

Furthermore, the higher viscosity slows the organic solvent diffusion into the aqueous phase because of the increased viscosity resistance to shear forces throughout the emulsification process. This results in the formation of coarse emulsions and, thus, larger nanoparticles [50,58].

Concerning the PVA concentration in IAP (X3), the nanoparticles’ size was significantly influenced by increasing the stabilizer concentration (*p* < 0.0001). PVA could be placed at the interface of the organic solution and the aqueous medium, thereby decreasing the interfacial tension while raising the net shear stress that promotes the production of small particles [50]. In contrast, increasing the PVA concentration in IAP increased the aqueous phase viscosity, and as a result of the lower shear stress, the mean diameter of the particles increased [59]. Moreover, increasing particle size with increasing stabilizer concentration might be based on the PVA molecules’ gelatinization because of the strong hydrogen bonding between inter- or intra-molecules of PVA via hydroxyl groups that cause the particle size to increase [52].

The literature indicates that a portion of PVA persists associated with nanoparticles as it forms an interconnected network with PLGA at the surface via attaching PVA hydroxyl groups to PLGA acetyl groups [18]. The proposed mechanism entails overlapping PVA and PLGA molecules, particularly during the evaporation of the organic solvent. Therefore, PVA’s hydrocarbon chains (hydrophobic segments) remain encapsulated in the polymeric matrix [57,60]. However, a high PVA concentration may result in the inappropriate orientation of these chains on the particles’ surface. Therefore, greater concentrations of residual PVA may cause the size of the particles to grow [59].

#### 3.1.3. Influence of Formulation Variables on PDI (Y3)

PDI is a ratio that provides insight into the uniformity of particle size distribution in a given system. Most researchers consider PDI values ≤0.5 to be acceptable [21]. PLGA NPs’ PDI values ranged between 0.331 ± 0.016 and 0.496 ± 0.010 (Table 2), indicating that the prepared PLGA NPs are relatively homogenous polydispersed systems [61].

Increasing the L/G ratio in PLGA 85:15 compared to PLGA 50:50 (X1), as well as increasing the PLGA wt (X2), significantly decreased PDI (*p* = 0.0003 and *p* < 0.0001, respectively). As aforementioned, the organic phase viscosity determines the average size of nanoparticles. This could be addressed such that by increasing L/G ratio or PLGA wt leads to the formation of more viscous droplets that are difficult to break down into smaller droplets, resulting in maintaining the size of nanoparticles within a narrow range, reducing the diversity in NPs size, and keeping low PDI values [55]. In contrast, as previously discussed, low Mwt PLGA polymer or PLGA wt produces nanoparticles with a smaller size. The consecutive droplets’ breakdown resulted in nano-droplets of various sizes, which by turn increased the PDI values [55].

The presence of PVA in the IAP (X3) resulted in a significant reduction in PDI values (*p* < 0.0001). This could be ascribed to PVA being adsorbed on the nanoparticles’ surface through the hydrogen and Van der Waals interactions with PLGA, which granted the particles’ stabilization by preventing their agglomeration and hence their uniform dispersion, as discussed before [56].

#### 3.1.4. Influence of Formulation Variables on ZP (Y4)

Zeta potential (ZP) provides an indication of the degree of stability of NPs systems [62]. Statistical analysis revealed that both L/G ratio (X1) and PLGA wt (X2) had a non-significant effect on ZP (*p* = 0.1066 and 0.8217, respectively). As cited in Table 2, the unmodified PLGA NPs exhibit a relatively low negative zeta potential ranging from −4.97 ± 0.33 mV to −8.81 ± 0.41 mV, which contributes to the enhancement of particles’ stability as the repulsive forces inhibit particles’ aggregation. This negative charge might be related to uncapped-end ionized carboxyl groups of PLGA molecules on PLGA NPs’ surfaces [51].

Contrarily, by increasing the concentration of the non-ionic stabilizer PVA in IAP (X3), the ZP values were significantly decreased (*p* < 0.0001). As previously stated, the interpenetration of PVA and PLGA molecules during nanoparticle formation has been proposed as previously discussed about PVA-PLGA binding. The existence of PVA resulted in the formation of a stable coating network on the polymer’s surface. This network shielded the surface charge, and the shear plane was moved away from the particle surface, resulting in slightly negative zeta potential values [60,63]. PVA cannot entirely shield this negative surface charge because of the carboxylic functional groups of PLGA [64]. Despite their relatively weak zeta potential, the nanoparticles are stabilized by the layers of PVA that surround them via steric hindrance [60]. These layers cannot be removed entirely despite repeated washing [57].

### 3.2. Optimization of CBPE-PLGA NPs

Response optimization was utilized to determine the combination of factors/levels that optimizes the nanoparticle property of interest during nanoparticle preparation. The response of nanoparticles was optimized to attain CBPE-PLGA NPs having the maximum EE% (highest priority) with minimum PS while keeping the PDI and ZP values in the desired range. Consequently, the optimal formula (F4), with EE% of 72.30 ± 2.86%, PS of 370.10 ± 10.31 nm, PDI of 0.398 ± 0.001, and ZP of −5.40 ± 0.21 mV was chosen with a desirability value (D) of 0.775. A direct correlation was identified between its observed and predicted values (Table 3).

### 3.3. Surface-Modification of the Optimum CBPE-PLGA NPs

In our study, CS-CBPE-PLGA NPs (CS-F4) were fabricated to achieve the best antitumor activity of CBPE by targeting colon cells and enhancing the PLGA NPs’ cellular permeation and uptake by modifying the surface charge of the optimum CBPE-PLGA NPs (F4).

ZP is an essential indicator for determining the surface charge of nanoparticles. Most cancer cell membranes have negatively charged surfaces; consequently, PLGA nanoparticles have a low affinity toward cancer cells because of the negatively charged carboxyl groups on the PLGA surface. Therefore, chitosan was used to alter the charge of the PLGA NPs’ surface of the optimum formula (F4) [65]. CS-F4 exhibit a ZP of 21.63 ± 2.46 mV, which is significantly different (*p* < 0.0001) than the ZP of the unmodified formula (F4), having a value of −5.40 ± 0.21 mV. The acquired positive charge of ZP was due to the amino groups in chitosan, which indicates the successful coating of PLGA NPs. The intermolecular hydrogen bonding of chitosan amino groups with PLGA carboxylic groups allows chitosan adsorption on the PLGA surface while hiding the PLGA carboxylic groups’ inherent negative charge [49,66]. It has been reported that the first monomolecular adsorption layer can be formed by electrostatic interaction between chitosan and the negatively charged PLGA NPs. The subsequent adsorption of chitosan would be driven by hydrogen bonding or Van der Waal’s force.

The higher Z-potential values of CS-PLGA NPs than those of uncoated PLGA NPs can result in a higher repulsive force; hence, higher resistance to aggregation is expected, leading to more stability of the formed particles [19,49]. As well, the particle size of CS-F4 increased significantly (*p* = 0.0026) to 417.67 ± 6.77 nm compared to the unmodified formula (F4, 370.10 ± 10.31 nm), possibly because of chitosan adsorption on the porous surface of PLGA NPs [67] resulting in an increase in the polymer content after addition of chitosan [68]. In addition, the viscosity was increased following chitosan, which lowers the shear stress during emulsion formation and causes a further enlargement in the average size of the prepared particles [21].

As discussed previously, the increase in size kept the NPs within a narrow range, reducing the heterogeneity and variability in their size, resulting in a lower PDI of 0.323 ± 0.019. This could be the reason for the significant PDI value reduction (*p* = 0.0027) after CS coating [55]. A non-significant difference in the EE% was observed in CS-F4 (74.74 ± 1.48%) compared to F4 (72.30 ± 2.86%), (*p* = 0.2605). It could be concluded that the increase in PS together with the positive ZP values confirmed the effective coating and the efficient surface modification of the optimum formula (F4) with chitosan [19].

### 3.4. In Vitro Characterization of the Optimized CBPE-PLGA NPs (F4) and CS-CBPE-PLGA NPs (CS-F4)

#### 3.4.1. TEM Examination

**Figure 2:**
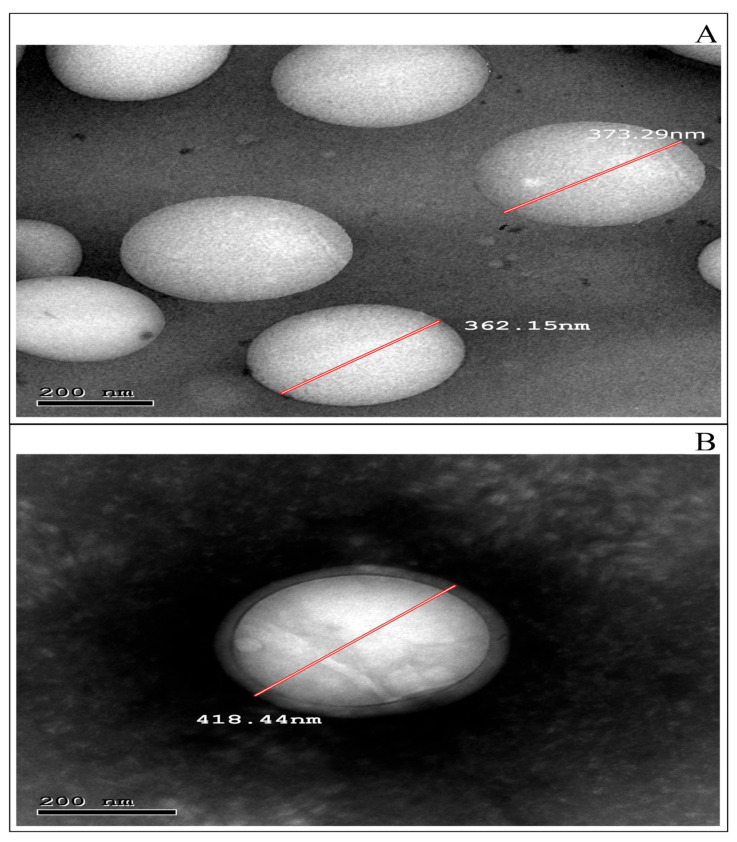
TEM photomicrographs of (**A**) optimum CBPE-PLGA NPs (F4) and (**B**) chitosan surface modified CBPE-PLGA NPs (CS-F4).

Figure 2A,B shows spherical nanoscopic non-aggregated particles for F4 and CS-F4, respectively. A solid, dense polymer center enveloped by an evenly distributed chitosan coat is represented in Figure 2B, confirming the complete coating of nanoparticles. The possible explanation for this finding is that coating with chitosan is caused by the potential binding ability to the surface of PLGA through hydrogen bonds formed between its protonated amino group and PLGA’s carboxylic groups [69]. Obviously, the observed PS of TEM was proportional to the particle size data provided by the Zetasizer (Malvern Instrument Ltd., Worcestershire, UK).

#### 3.4.2. ATR-FTIR Examination

**Figure 3:**
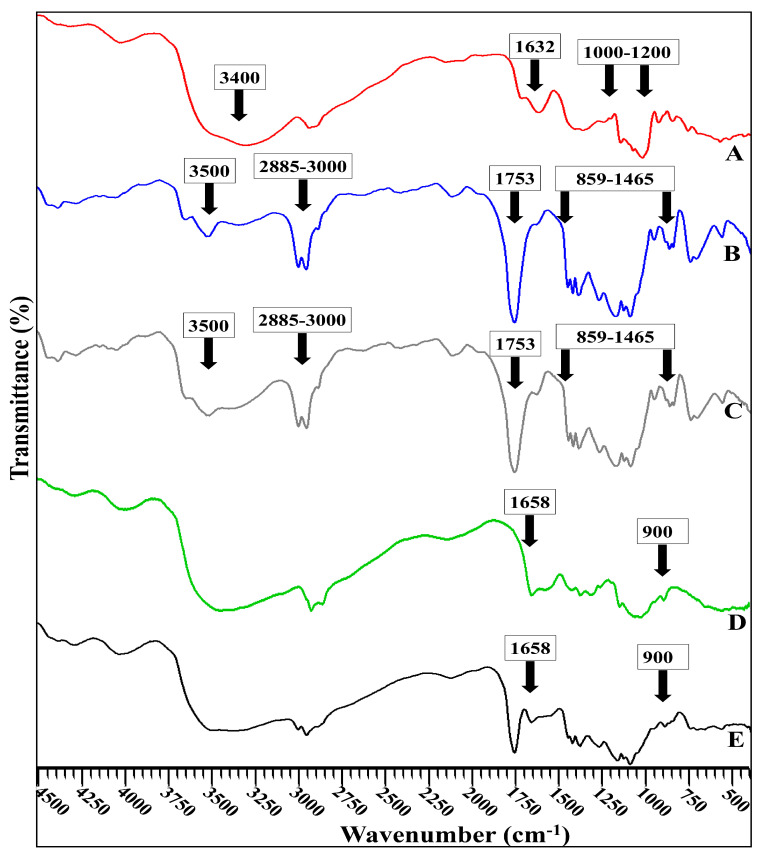
ATR-FTIR spectrum of: (**A**) CBPE, (**B**) blank PLGA NPs (F4), (**C**) CBPE-PLGA NPs (F4), (**D**) chitosan, and (**E**) CS-CBPE-PLGA NPs (CS-F4).

Figure 3 depicts FT-IR spectra of the optimum formula F4 and CS-F4 compared with free CBPE, CS, and blank formulation of (F4). The ATR-FTIR spectra were investigated to evaluate any possible interaction between PLGA and CBPE during CBPE encapsulation into PLGA NPs. ATR-FTIR spectra were also used to analyze the surface chemical composition of PLGA NPs and CS chains, confirming the coating of the PLGA NPs with CS.

The FTIR spectrum of CBPE (Figure 3A) shows the phenolic OH at 3400 cm^−1^ [70], C=O stretching phenyl ring amino acid at 1632 cm^−1^ [71], and the stretching vibration of the glycoside bond (C–O–C) which appears in the region of 1000–1200 cm^−1^ [72].

The blank formulation spectrum of F4 (Figure 3B) exhibits stretching peaks of C=O at 1753 cm^−1^ and C–H bending at 859–1465 cm^−1^, as well as CH, CH_2_, and CH_3_ stretching vibration between 2885 and 3000 cm^−1^, and finally OH stretching around 3455–3500 cm^−1^. The blank formulation also responded at 2955 cm^−1^ because of the linear CH_2_ stretching and 1756 cm^−1^ caused by the ester bond. These bands are reported to be characteristic of PLGA [49].

The distinguishing bands of CBPE did not exist in the FTIR spectrum of F4 (Figure 3C). This might indicate the proper encapsulation of drug extract into the core of the PLGA NPs [21,64].

According to Figure 3D, the FTIR spectrum of CS exhibits intense peaks at 1658 cm^−1^, confirming the presence of amide I. In addition, the band characteristic for saccharin stretching vibration appears at 900 cm^−1^ [49].

The distinctive bands of CS are noticed in the FT-IR spectrum of CS-CBPE-PLGA NPs alongside the characteristic bands of PLGA (Figure 3E), suggesting the existence of chitosan coating on the surface of the optimum CBPE-PLGA NPs (F4). These findings are per the previously published research articles [21,23,67].

#### 3.4.3. In Vitro CBPE Release Study

**Figure 4:**
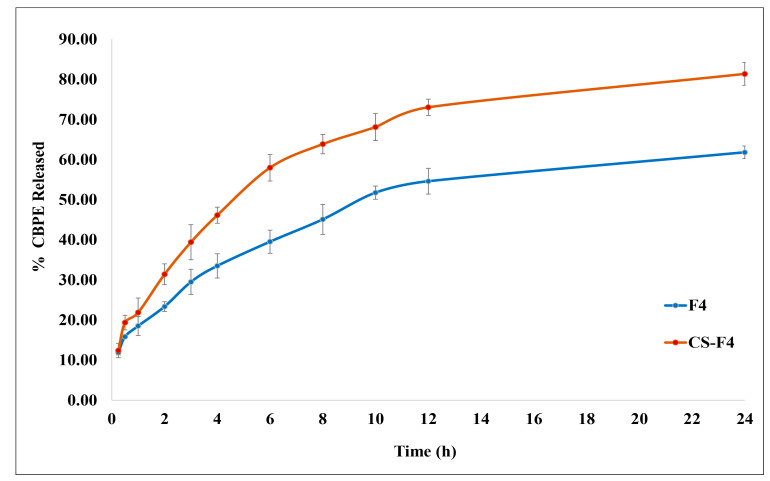
In vitro release profile of CBPE from the optimum CBPE-PLGA NPs (F4) and CS-CBPE-PLGA NPs (CS-F4).

Figure 4 depicts the in vitro release characteristics of CBPE from the optimum formula F4 and CS-F4. Both formulae manifested a biphasic release profile, with an initial burst release, where 11.89% ± 0.61 and 12.39% ± 1.80 of CBPE were released, respectively, in the first 15 min, followed by a sustained release pattern that persisted until the end of the study, which would be appropriate for a prolonged drug effect.

It is known that the *T_g_* influences the initial burst release [73]. In a matter of minutes, water could be absorbed into the core of PLGA NPs [74]. As PLGA polymers absorb water and surfactants that act as plasticizers, their *T_g_* temperature decreases by 15 °C [73]. Therefore, the *T_g_* of the formulation prepared with (PLGA 50:50, the polymer with the lowest *T_g_* value) will be close to 37 °C (the release temperature), rapidly compared to the higher *T_g_* polymer (PLGA 85:15). That means, as discussed before, PLGA 50:50 attained the equilibrium state rapidly (physical aging), which resulted in the formation of microvoids because of the relaxation process. These microvoids enhanced more water absorption, which dissolved the unentrapped hydrophilic drug (CBPE), which released faster with a significant initial burst effect [75]. Lappe et al. [76] showed that only the drug absorbed on the nanoparticle surface caused a burst release when the release medium temperature was close to the *T_g_* of the nanoparticles.

The slower drug release phase may be correlated to the drug impeded in the PLGA polymeric matrix that is slowly diffused out because of the PLGA polymer’s hydrophobic nature, which makes it harder for water to get into the polymeric matrix. It may be owing to the more extended diffusion pathway of the cranberry molecules located in the core of the PLGA matrix, followed by the bioerosion of the polymer [43].

It is noticeable that coating the PLGA NPs with chitosan in CS-F4 increased the overall drug release rate markedly compared to the uncoated one (F4). The calculated similarity factor “*f_2_*” confirmed that the release profile of CBPE from CS-F4 is significantly different than that from F4, where “*f_2_*” = 43.29. This could be explained by the fact that CS is more hydrophilic than PLGA, leading to a greater degree of water permeability at the CS surface, resulting in higher hydration of the PLGA NPs matrix and enhancing the drug diffusion, consequently enhancing CBPE release [13,77].

Results revealed that in vitro CBPE release from both F4 and CS-F4 follows the Higuchi release, where R^2^ values were 0.964 and 0.941, respectively. The “*n*” values of 0.3147 and 0.4201 for the Korsmeyer–Peppas model were used to expect the mechanism of CBPE release and indicated that CBPE release was controlled by Fickian diffusion (i.e., erosion and diffusion via the polymeric matrix) [18].

#### 3.4.4. Ex Vivo Permeation Study

**Figure 5:**
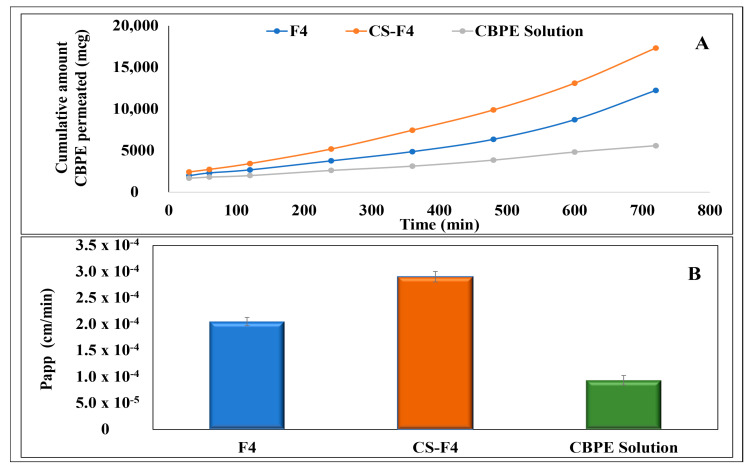
Ex vivo permeation parameters of CBPE: (**A**) cumulative amount of CBPE permeated (mcg) ± SD from the optimized CBPE-PLGA NPs (F4) and CS-CBPE-PLGA NPs (CS-F4) compared to CBPE solution and (**B**) permeation coefficient (*P*_app_ ± SD) from the optimized CBPE-PLGA NPs (F4) and CS-CBPE-PLGA NPs (CS-F4) compared to CBPE solution.

Figure 5 depicts the permeation flux (both amount permeated and permeability coefficient) of CBPE from F4 and CS-F4 compared to CBPE solution across the ileum. Figure 5A demonstrates that the cumulative amount permeated of CBPE from F4 and CS-F4 shows better results than that of the CBPE solution at all different intervals. It is revealed through Figure 5B that the permeability coefficient of both CBPE formulae was significantly increased by 2.19- and 3.10-fold, with permeability coefficients (*P*_app_) equal to 2.05 × 10^−4^ cm/min and 2.91 × 10^−4^ cm/min for F4 and CS-F4, respectively, compared to the CBPE solution (9.36 × 10^−5^ cm/min).

The significant improvement in CBPE permeability from the prepared PLGA NPs could be related to the hydrophobic nature of PLGA polymer, which could improve particle interaction with the lipophilic cell membranes, resulting in increased cellular uptake of CBPE-PLGA NPs through enterocytes and M-cells of Peyer’s patches compared to CBPE solution, which is characterized by high hydrophilicity and molecular weight, hindering its permeation [43].

Additionally, PVA is a non-ionic surfactant widely employed as a promising PLGA NPs stabilizer [78]. It was reported that surfactants could boost the transcellular transport of drugs by disturbing the lipid bilayer structural integrity of intestinal cell membranes, reducing mucus viscosity, increasing membrane fluidity, and promoting hydrophilic drugs’ paracellular transport [79]. Furthermore, incorporating PVA, a bioadhesive substance, can also be effective to promote the uptake of nanoparticles by intestinal enterocytes. Adherence of a nanocarrier system to mucus may increase drug contact time and interaction with the underlying epithelium, thereby increasing intestinal permeability. [51].

CS-F4 exhibited a significant enhancement in the permeability flux by 1.42-fold compared to F4. This might be attributed to chitosan’s mucoadhesive characteristics. Chitosan mucosal adhesion is achieved by the protonation of the amino group, which makes the chitosan macromolecule positively charged, providing better interaction between the nanoparticles and the intestinal epithelium [23,31]. Consequently, a rise in drug concentration at the absorption site increases its para- or transcellular permeation. The higher drug release from CS-F4 also confirmed this finding compared to the uncoated F4 [31,77].

#### 3.4.5. Stability Study

Statistical analysis indicated a non-significant alteration in physical appearance, EE%, PS, PDI, and ZP after three months of storage at 4 °C. These data revealed that F4 and CS-F4 were stable under the specified terms. The stability of uncoated PLGA NPs is most likely owing to the steric repulsion between particles attributed to the presence of PVA as stabilizer. Regarding CS-F4, the repulsive forces are induced by the high positive surface charge imparted by the chitosan coating [77].

#### 3.4.6. In Vitro Antitumor Activity

##### Cytotoxic Activity Determination Assay (IC_50_)

The cytotoxic activities of the CBPE solution, CBPE-PLGA NPs (F4), and CS-CBPE-PLGA NPs (CS-F4) on the HT-29 cell line were evaluated by determining IC_50_ values, which showed that all of them had cytotoxic effects against HT-29 cell lines. Interestingly, the CS-F4 formula had the highest significant (*p* < 0.05) cytotoxic activity (IC_50_ = 1 µg/mL) compared to F4 and the CBPE solution, which had IC_50_ of 1.75µg/mL and 2.6µg/mL, respectively.

##### Effect on Caspase-3 Protein Level

**Figure 6:**
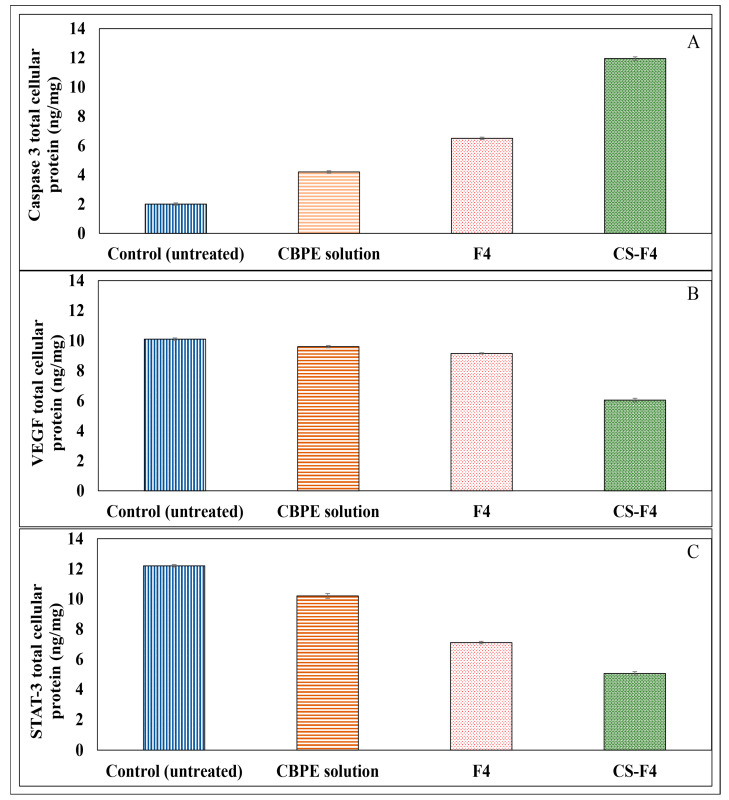
Effects of CBPE solution, CBPE-PLGA NPs (F4), and CS-CBPE-PLGA NPs (CS-F4) on (**A**) caspase-3 protein level (ng/mg tissue protein), (**B**) VEGF protein level (ng/mg tissue protein), and (**C**) STAT-3 protein level (ng/mg tissue protein) in HT-29 cells. All treatments were given for three days. Results are presented as mean ± S.E.M., (*n* = 4).

Figure 6A shows that the CS-F4 formula exerted a significant caspase-3 protein level expression stimulation compared to control, CBPE solution, and F4 in the HT-29 cell lines (*p* < 0.05). However, F4 showed a moderate increase in caspase-3 protein level induction compared to CBPE solution. So, both formulae (F4 and CS-F4) could have good apoptotic activities.

##### Effect on VEGF Protein Level

Figure 6B shows that the CS-F4 formula provided the highest significant VEGF protein level inhibition in the HT-29 cell line compared to control, CBPE solution, and F4 (*p* < 0.05). Accordingly, CS-F4 revealed high cytotoxic activity compared to the uncoated one (F4).

##### Effect on STAT-3 Protein Level

Intriguingly, the CS-F4 formula showed the most significant STAT-3 protein level inhibition (*p* < 0.05) and, thus, the highest cytotoxicity among the tested formulae in the HT-29 cell line as compared to the control, as illustrated in Figure 6C.

Based on the previous results, CS-CBPE-PLGA NPs (CS-F4) exhibited the most significant caspase-3 protein level expression stimulation and inhibition of both VEGF and STAT-3 protein expression levels compared to control, CBPE solution, and uncoated CBPE-PLGA NPs (F4). Therefore, chitosan surface-modified PLGA NPs could be a promising delivery system to promote CBPE permeation and uptake via intestinal endocytosis, thereby boosting colon cells targeting and cytotoxicity against colon cancer.

## 4. Conclusions

Chitosan surface-modified PLGA NPs have been successfully fabricated via a modified double-emulsion (W/O/W) solvent-evaporation technique for the encapsulation of CBPE aiming to enhance its intestinal permeability and antitumor activity against colon cancer. The prepared CS-F4 PLGA NPs showed spherical positively charged particles (ZP of +21.63 ± 2.46 mV) with acceptable size (417.67 ± 6.77 nm) and relatively good EE% (74.74 ± 1.48%). CBPE release from CS-F4 follows a Higuchi release pattern with a significant enhancement in the intestinal permeability (3.10-fold) with a permeability coefficient of 2.91 × 10^−4^ cm/min compared to free CBPE solution (9.36 × 10^−5^ cm/min). Furthermore, in vitro studies established the superiority of CS-F4 compared to the CBPE solution in boosting the cytotoxic activity against HT-29 colon cancer cells confirmed by the lowest IC_50_ of 1 µg/mL), the most significant stimulation of caspase-3 protein level expression (11.95 ± 0.12 ng/mg) and suppression of VEGF (6.05 ± 0.10 ng/mg), as well as STAT-3 (5.05 ± 0.14 ng/mg) protein expression levels. Based on these findings, it is possible to suggest that chitosan surface-modified NPs loaded with CBPE could be a promising herbal-based nanocarrier system for targeting colon cancer cells.

## Figures and Tables

**Table 1:** 2^3^ full factorial design for preparing CBPE-PLGA NPs formulae.

Factors (Independent Variables)	Levels	
X1: L/G ratio	50:50	85:15
X2: PLGA Weight (mg)	20	50
X3: PVA Conc in IAP (%)	0	1
**Responses (Dependent Variables)**	**Desirability Constraints**	
Y1: EE%	Maximize	
Y2: PS (nm)	Minimize	
Y3: PDI	In the range	
Y4: ZP	In the range	

CBPE means cranberry powdered extract; CBPE-PLGA NPs: CBPE-loaded PLGA nanoparticles; PLGA: poly lactide co-glycolic acid; L/G: lactide/glycolide; IAP: internal aqueous phase; EE%: entrapment efficiency percentage; PS: particle size; PDI: polydispersity index; and ZP: zeta potential.

**Table 2:** Composition and in vitro characterization of the prepared CBPE-PLGA NPs.

Formulations	X1 L/G Ratio	X2 PLGA Weight (mg)	X3 PVA Conc in IAP (%)	Y1 EE%	Y2 PS (nm)	Y3 PDI	Y4 ZP (mV)
F1	PLGA 50:50	20	0	48.30 ± 2.21	259.87 ± 4.68	0.496 ± 0.01	−8.81 ± 0.41
F2	PLGA 50:50	50	0	56.82 ± 2.36	345.10 ± 9.17	0.431 ± 0.040	−8.57 ± 0.48
F3	PLGA 50:50	20	1	58.38 ± 2.57	284.87 ± 8.11	0.453 ± 0.048	−4.98 ± 0.25
F4	PLGA 50:50	50	1	72.30 ± 2.86	370.10 ± 10.31	0.398 ± 0.001	−5.40 ± 0.21
F4-CS	PLGA 50:50	50	1	74.74 ± 1.48	417.67 ± 6.77	0.323 ± 0.019	+21.63 ± 2.46
F5	PLGA 85:15	20	0	41.95 ± 2.84	265.87 ± 4.15	0.481 ± 0.001	−8.32 ± 0.56
F6	PLGA 85:15	50	0	51.61 ± 2.16	365.90 ± 6.79	0.406 ± 0.025	−7.13 ± 0.94
F7	PLGA 85:15	20	1	53.12 ± 2.72	417.67 ± 6.77	0.379 ± 0.007	−4.97 ± 0.33
F8	PLGA 85:15	50	1	57.83 ± 1.99	423.10 ± 8.36	0.331 ± 0.016	−5.78 ± 0.74

Each formula contains the same quantity of CBPE (30 mg/mL). Data are provided as mean ± SD, (*n* = 3). CBPE means cranberry powdered extract; CBPE-PLGA NPs: CBPE-loaded PLGA nanoparticles; PLGA: poly lactide co-glycolic acid; L/G: lactide/glycolide; IAP: internal aqueous phase; EE%: entrapment efficiency percentage; PS: particle size; PDI: polydispersity index; ZP: zeta potential.

**Table 3:** (A) 2^3^ factorial analysis outcomes of CBPE-PLGA NPs formulae and (B) observed and predicted outcomes of the optimum formula (F4).

(A) Responses	R^2^	Adjusted R^2^	Predicted R^2^	Adequate Precision	Significant Factors
EE (%)	0.921	0.893	0.842	17.99	X1, X2, X3
PS (nm)	0.952	0.935	0.905	21.83	X1, X2, X3
PDI	0.861	0.812	0.724	12.86	X1, X2, X3
ZP (mV)	0.914	0.884	0.828	13.71	X3
**(B) Response**		**Y1 ** **EE%**	**Y2 ** **PS**	**Y3 ** **PDI**	**Y4 ** **ZP**
Observed values		72.30	370.10	0.398	−5.40
Predicted values		71.01	358.28	0.4	−5.56

CBPE: cranberry powdered extract; CBPE-PLGA NPs: CBPE-loaded PLGA nanoparticles; PLGA: poly lactide co-glycolic acid; EE%: entrapment efficiency percentage; PS: particle size; PDI: polydispersity index; ZP: zeta potential.

## Data Availability

Data are contained within the article and the Supplementary Materials.

## Supplementary Materials

The following supporting information can be downloaded at https://www.mdpi.com/article/10.3390/pharmaceutics15020606/s1: Materials, Kits, Cell line, and Instruments.
