# Supplementary material for: Chitosan Surface-Modified PLGA Nanoparticles Loaded with Cranberry Powder Extract as a Potential Oral Delivery Platform for Targeting Colon Cancer Cells
Source: Pharmaceutics. 2023 Feb 10;15(2):606. doi: 10.3390/pharmaceutics15020606 (PMC9964659; doi:10.3390/pharmaceutics15020606)
Supplement: Supplementary file 1 [file pharmaceutics-15-00606-s001.zip › pharmaceutics-2159109-supplementary.pdf]

pharmaceutics

# Chitosan surface-modified PLGA nanoparticles loaded with cranberry powder extract as a potential oral delivery platform for targeting colon cancer cells

**Mona M. Mostafa [1], Maha M. Amin [2,*], Mohamed Y. Zakaria [3,4], Mohammed Abdalla Hussein [5], Marium M. Shamaa [6] and Shady M. Abd El-Halim [1,*]**

1 Department of Pharmaceutics and Industrial Pharmacy, Faculty of Pharmacy, October 6 University, 6th of October City, Giza 12585, Egypt; mona.mohammed.ph@o6u.edu.eg
2 Department of Pharmaceutics and Industrial Pharmacy, Faculty of Pharmacy, Cairo University, Cairo 11562, Egypt
3 Department of Pharmaceutics and Industrial Pharmacy, Faculty of Pharmacy, Port Said University, Port Said 42526, Egypt; mohamed.zakaria@pharm.psu.edu.eg
4 Department of Pharmaceutics and Industrial Pharmacy, Faculty of Pharmacy, King Salman International University, Ras Sudr 46612, South Sinai, Egypt
5 Faculty of Applied Health Sciences Technology, October 6 University, 6th of October City, Giza 12585, Egypt; prof.husseinma@o6u.edu.eg
6 Biochemistry Department, Clinical and Biological Sciences Division, College of Pharmacy, Arab Academy for Science, Technology and Maritime Transport, Alexandria 1029, Egypt; marium.muhamed@aast.edu
* Correspondence: maha.amin@pharma.cu.edu.eg (M.M.A.); shady_mohammed@o6u.edu.eg (S.M.A.E.-H.); Tel.: +20-1221602540 (M.M.A.); +20-1119994874 (S.M.A.E.-H.)

## 1. Materials

Cranberry powder extract (CBPE), PLGA (85/15 DL-lactide/Glycolide copolymer, Mwt: 50.000–75.000, inherent viscosity 0.55–0.75 dL/g, 0.1% (w/v) in chloroform (25°C) and PLGA (50/50 DL-lactide/Glycolide copolymer, Mwt: 24.000–38.000, inherent viscosity 0.32–0.44 dL/g, 0.1% (w/v) in chloroform (25°C), chitosan (CS, low molecular weight 50.000–190.000 Da, 75–85% deacetylated), Polyvinyl alcohol (PVA, Mw = 85.000–124.000, +99% hydrolyzed), Dichloromethane (DCM), and methanol were purchased from Sigma-Aldrich Chemical Co., St Louis, MO, USA. Deionized water was prepared using (Purite Water Purification Systems, Select HP, Suez, Birmingham UK). Sodium chloride, Potassium chloride, Sodium phosphate dibasic, and Potassium phosphate monobasic were purchased from Adwic-El Nasr Pharmaceutical Co., Cairo, Egypt. Chemicals purchased from Sigma-Aldrich are of a highly pure grade. All other chemicals used are of analytical grade.

## 2. Kits and cell line

Sigma in vitro assay kit MTT based (Sigma-Aldrich Chemical Co., St Louis MO, USA). Caspase 3 activation assay kit (Abcam, Cambridge, UK, Catalog # ab281235). Vascular Endothelial Growth Factor (VEGF) protein level assay kit (Abcam, Cambridge, UK, Catalog # ab209882). Signal transducer and activator of transcription-3 (STAT-3) assay kit (Abcam, Cambridge, UK, Catalog # ab126459).

Colon cancer cell line (HT-29), obtained from American Type Culture Collection ATCC (St Louis, MO, USA), were cultured using Dulbecco's Modified Eagle Media (DMEM, Invitrogen/Life Technologies, Thermo Fischer Scientific, Basel, Switzerland) supplemented with 10% Fetal Bovine Serum (FBS, Sigma-Aldrich Chemical Co., St Louis MO, USA), and 1% penicillin-streptomycin. All other chemicals and reagents were supplied from Sigma-Aldrich Co.

## 3. Instruments

Rotary-evaporator (Heidolph VV 2000, Burladingen, Germany). Probe sonication with an ultrasonic processor (Chrom Tech, model UP-500, Crowborough, UK). Cooling centrifuge (Sigma-Aldrich 3–30 KS, Hamburg Germany). Centrifugal filter units (Amicon® Ultra-4, 3K, Millipore, Germany). UV/VIS spectrophotometer (Shimadzu, model UV-1601 PC, Kyoto, Japan), FTIR (IRaffinity-1 Spectrometer, Shimadzu, Kyoto, Japan) fitted with ATR-8200HA (Attenuated Total Reflectance Unit) accessory. HPLC (LC-20AD liquid chromatograph, Shimadzu, Japan) consisting of two LC-10ADvp pumps, a DGU-20A continuous degassing unit equipped with an SIL-20A autosampler and an SPD-20A UV–vis detector. Chromatographic separations were achieved by a Pronto SIL® RP-C18 (150 × 4.6 mm, 5 μm) column (type SC-150, Bischoff Chromatography, Berlin, Germany). HPLC data were acquired and processed using LC solution Software, version 1.25 SP4 (Shimadzu, Kyoto, Japan). Malvern Zetasizer (Malvern Instrument Ltd., Worcestershire, UK). Hot-plate magnetic stirrer (7280; Ugo Basile, Italy). Reciprocal shaking water bath (Model 25, Precision scientific group, Chicago, USA). Transmission Electron Microscopy (TEM, JEOL, JEM-1230, Tokyo, Japan).
